# On the Use of Gutta Percha in Fractures of the Jaw

**Published:** 1851-01

**Authors:** H. N. Wadsworth

**Affiliations:** Georgetown, D. C.


### ARTICLE X

### *On the Use of Gutta Percha, in Fractures of the Jaw.* By H. N. WADSWORTH, Georgetown, D. C.

ABOUT six weeks since, E. O., a lad of this city, of ten years of age, in running across the street, fell and striking his face against the curbstone injured his jaw and teeth severely: a physician passing, was called in and finding the injury confined to the mouth, advised sending for me.

On examination I found the left temporary inferior cuspid broken off within a quarter of an inch of its apex, and thrown upon the pavement; the process anterior to this tooth split down and turned outwards, with the lateral and central permanent inferior incisors: the superior central and lateral permanent incisors; the temporary cuspid and the two temporary molars of the left side with the process on their labial or external side were doubled into the mouth in a single piece ; the process appeared to break near the points of the roots of these teeth, and with the exception of a few fragments near the incisors was connected and formed a solid piece with the teeth attached; the process on the palatine side of the teeth, did not seem to be injured; the points of the teeth, or cutting edges, were fully half an inch out of their proper position. Dr. Riley, the family physician, being called in at my request; after a careful examination, it was decided to endeavor to save the bone and the teeth attached, as the extraction of the teeth would have brought the whole piece of process with it; at my suggestion the following plan was adopted, and left to me to execute by the politeness of Dr. Riley.

Extracted the last superior temporary molar of the left side, it being very carious : fitted a gutta percha mould to the teeth that were displaced in the upper jaw : tied a ligature around the two teeth in the lower jaw, and drawing them to their place with the bone attached, fastened the ligature around the lower temporary molar of the right side ; fitted a gutta percha mould over the lower teeth; cutting out enough to avoid touching the two fractured inferior teeth ; caused these two moulds to antagonise so as to give the upper one an upward and outward pressure when the jaws were closed ; having previous to all this removed the small pieces of bone, and washed the parts well in cold water, the moulds were applied, the jaws closed, and a bandage passed tightly around the chin and over the head; and cold applications of water ordered to be constantly applied to the face.

No medicine of any kind given ; a light liquid diet; and two or three injections to move the bowels ; on the second night we decided to leave off the moulds during the night, owing to the tumefied state of the adjacent parts, but by constant application of cold water it was so much reduced as to render it proper to resume them, and with the exception of their removal to allow him to eat and wash the wound, which was done with a syringe and cold water, it was worn for three days when it was discontinued, the bone having set. No evidence of the accident now exists, except the elongation of the superior teeth about two lines beyond their fellows.

About two or three years ago, a man was sent to my office to have his lower jaw set: twelve hours previous he had been struck by a "slung-shot," at the angle of the lower jaw, with such force as to break it at the ramus of the left side, and again at a point very near the mental foramen of the right side: the whole of this part of the inferior maxillary was detached, and had fallen as far as the soft parts would permit: obtaining the assistance of Dr. Charles H. Cragin, we proceeded to fit a gutta percha mould to his jaw, which, when it had become hard, enabled us to confine it in the most perfect manner, with bandages passed over the head. This man being a captain of a vessel, belonging in Massachusetts, and leaving the same day, I have never heard from him since.

These two cases are reported to call the attention of the profession, to the usefulness of gutta percha in setting fractures of bones.

